# Optimization of Regularization Parameters in Compressed Sensing of Magnetic Resonance Angiography: Can Statistical Image Metrics Mimic Radiologists' Perception?

**DOI:** 10.1371/journal.pone.0146548

**Published:** 2016-01-08

**Authors:** Thai Akasaka, Koji Fujimoto, Takayuki Yamamoto, Tomohisa Okada, Yasutaka Fushumi, Akira Yamamoto, Toshiyuki Tanaka, Kaori Togashi

**Affiliations:** 1 Department of Diagnostic Imaging and Nuclear Medicine, Graduate School of Medicine, Kyoto University, Kyoto, Japan; 2 Department of Systems Science, Graduate School of Informatics, Kyoto University, Kyoto, Japan; The Lee Kong Chian School of Medicine, SINGAPORE

## Abstract

In Compressed Sensing (CS) of MRI, optimization of the regularization parameters is not a trivial task. We aimed to establish a method that could determine the optimal weights for regularization parameters in CS of time-of-flight MR angiography (TOF-MRA) by comparing various image metrics with radiologists’ visual evaluation. TOF-MRA of a healthy volunteer was scanned using a 3T-MR system. Images were reconstructed by CS from retrospectively under-sampled data by varying the weights for the L1 norm of wavelet coefficients and that of total variation. The reconstructed images were evaluated both quantitatively by statistical image metrics including structural similarity (SSIM), scale invariant feature transform (SIFT) and contrast-to-noise ratio (CNR), and qualitatively by radiologists’ scoring. The results of quantitative metrics and qualitative scorings were compared. SSIM and SIFT in conjunction with brain masks and CNR of artery-to-parenchyma correlated very well with radiologists’ visual evaluation. By carefully selecting a region to measure, we have shown that statistical image metrics can reflect radiologists’ visual evaluation, thus enabling an appropriate optimization of regularization parameters for CS.

## Introduction

Time—of—flight (TOF) magnetic resonance angiography (MRA) has held its established position in routine brain MRI examinations owing to its non-invasiveness and excellent diagnostic ability of arterial stenoses and aneurysms[1]. Although its diagnostic ability has been shown to be comparable to computed tomography angiography (CTA)[2,3] as early as 2001, its major weaknesses include long scan time and limited spatial resolution relative to CTA[1,4].

On the other hand, ever since the first magnetic resonance (MR) images were acquired in the early 1970s, the demand for faster acquisition and higher resolution of MR images has never ceased. In the past decades we have seen dramatic advancements in hardware such as higher magnetic fields, faster switching gradients and phased-array coils, as well as progression in software including numerous pulse sequences and parallel imaging reconstruction techniques[5,6]. However, in the case of TOF-MRA, we have seen a mere two to three fold acceleration in acquisition time in the past decade albeit these technological advancements.

Recently the idea of compressed sensing (CS) has gained rapid momentum[7]. CS is a framework to recover an image by exploiting the underlying sparsity of the image in the appropriate transform domain. It minimizes a cost function:

$$E\left(x\right)=\;∥F\left(x\right)-y{∥}_{2}^{2}+\lambda ∥\Psi \left(x\right){∥}_{1}$$ (1)

where *y* is the acquired k-space data, *x* is the image to recover, *F* is the encoding matrix incorporating coil sensitivities and*Ψ* is a sparsifying transform such as wavelet. Since *E(x)* is convex, Eq (1) converges to an optimal solution for *x* by an adequate minimization algorithm such as A Fast Iterative Shrinkage-Thresholding Algorithm (FISTA) or nonlinear conjugate gradient (NLCG) algorithm. To achieve high incoherence between the image and data acquisition, the data in k-space was under-sampled in a pseudo-random fashion in the frequency domain. By appropriately making use of sparsity, incoherence, and nonlinear reconstruction, CS may allow an acceleration (under-sampling) factor of more than five with acceptable compromise in image quality[8].

CS reconstruction requires optimization of the weights for regularization parameters. As measures for evaluating quality of reconstructed images, statistical metrics such as normalized mean square error (NMSE), peak signal-to-noise ratio (PSNR) and structural similarity (SSIM) have been utilized[9]. Since these metrics measure distance, a gold standard data is required for calculation. Another approach is the L-curve or the L-surface method, which tries to find a balancing point between data fidelity and prior knowledge[10], in which case gold standard data is not required. Other than these quantitative metrics, there are a number of reports where optimal parameters are determined ‘empirically’ or by visual evaluation[11].

Regardless of the approach, the optimal parameters should be determined by the quality of the resultant images based on clinical significance. However, to date, research trying to shed light on the relationships between the optimization approach and clinical significance is very limited. The aim of this study was to establish a method that could determine the optimal weights for regularization parameters in CS of TOF-MRA by comparing various image metrics with radiologists’ scores for clinical significance.

## Materials and Methods

### MR imaging (data acquisition)

Study protocols were approved by the local ethics committee. With institutional review board approval by our institution (Kyoto University Hospital) and written informed consent, a healthy volunteer was scanned using a 3T-MR system (Vantage, TOSHIBA MEDICAL SYSTEMS CORPORATION, Otawara, Japan) with a 32-channel head coil for 3D TOF-MRA (TR/TE = 20/3.4ms, FA = 15°, matrix = 256 × 256 × 110, voxel size = 0.8 × 0.8 × 1.0 mm^3^). Parallel imaging or other under-sampling methods were not applied for the data acquisition. The MR scanning took 10 minutes and 15 seconds.

### Compressed sensing image reconstruction

**Fig 1:**
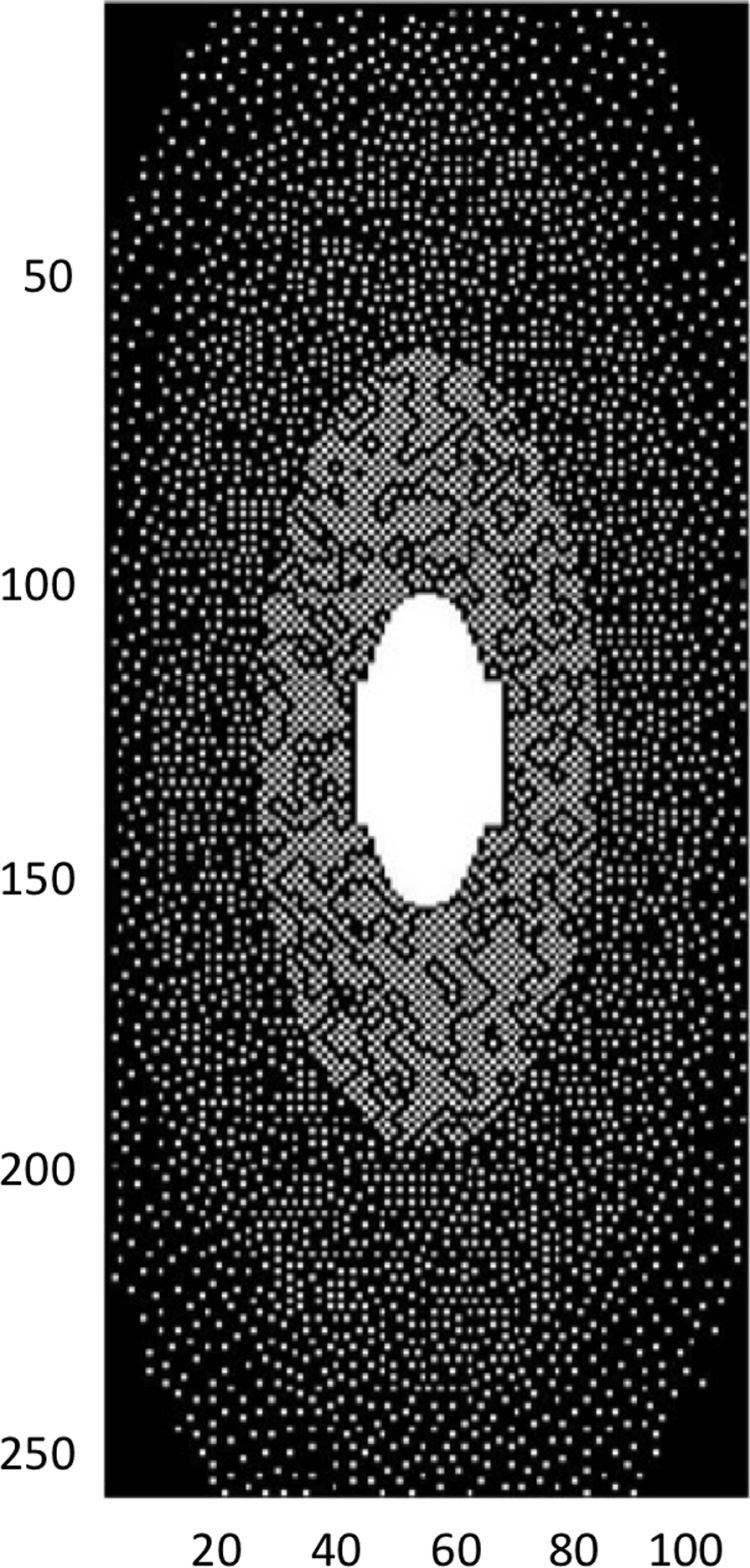
Variable density Poisson disk pattern under-sampling k-space mask. The sampling rate is 21% (acceleration rate is 4.7×). The mask is 256×110 pixels in resolution. The two axes are the two phase-encoding directions, namely transverse and craniocaudal. The frequency encoding direction is antero-posterior, and is continuous (not depicted).

**Fig 2:**
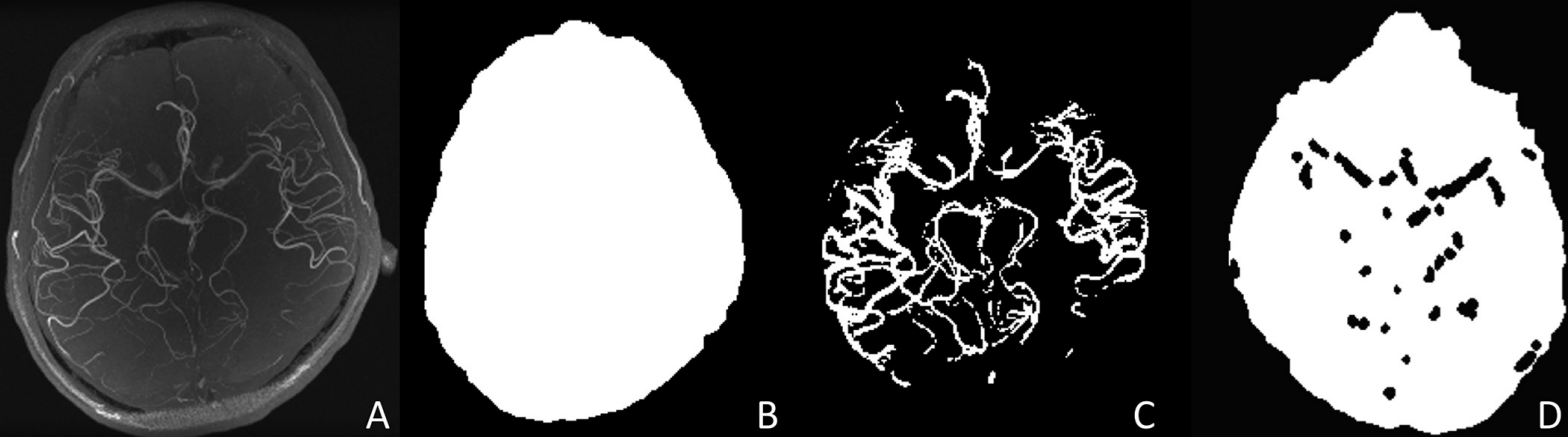
Craniocaudal maximum intensity projection images of the sum of squares images and brain masks. (A) Sum of squares image, (B) brain mask, (C) arterial mask and (D) an axial slice of the parenchymal mask.

Data were retrospectively under-sampled with a rate of 21% (4.7× acceleration) by using a variable-density Poisson disk pattern (Fig 1). The sum of squares (SoS) image was used as the reference standard (Fig 2).

After reducing the data size by a coil-compression technique[12], the fast composite splitting algorithm (FCSA)[13] was adopted for CS reconstruction in order to solve the following minimization problem:

$$\underset{x}{\text{min}}\left\{\frac{1}{2}{\left\|y-Ax\right\|}_{2}^{2}+\alpha {\left\|\Psi (x)\right\|}_{1}+\beta {\left\|TV(x)\right\|}_{1}\right\}$$ (2)

where *x* is the reconstructed image, *y* is the measured k-space data, *Ψ* and *TV* represent wavelet transform and total variation respectively, *A* represents the under-sampled Fourier transform, and *α* and *β* are parameters which control the weights for the sparsifying transformations considered (i.e. *Ψ* and *TV*).

FCSA is a fast and accurate image reconstruction algorithm which decomposes the hard composite regularization problem (2) into two simpler regularization sub-problems by a) splitting variable *x* into *x*_*1*_ and *x*_*2*_, b) performing operator splitting to minimize total variation regularization and L1 norm regularization sub-problems over *x*_*1*_ and *x*_*2*_, and c) obtaining the solution *x* by linear combination of *x*_*1*_ and *x*_*2*_. The algorithm is further accelerated with an additional step motivated by the effective acceleration scheme in Fast Iterative Shrinkage-Thresholding Algorithm (FISTA).

The value of *α* was varied with a range of {3.2 × 10^−3^, 1.0 × 10^−3^, 3.2 × 10^−4^, 1.0 × 10^−4^, 3.2 × 10^−5^, 1.0 × 10^−5^, 3.2 × 10^−6^, 1.0 × 10^−6^, 3.2 × 10^−7^, 1.0 × 10^−7^} and *β* with a range of {3.2 × 10^−3^, 1.0 × 10^−3^, 3.2 × 10^−4^, 1.0 × 10^−4^, 3.2 × 10^−5^, 1.0 × 10^−5^, 3.2 × 10^−6^, 1.0 × 10^−6^, 3.2 × 10^−7^, 1.0 × 10^−7^}; therefore a total of 100 CS reconstructions were performed. The number of iterations for each CS reconstruction process was empirically determined as 15. Calculation was performed by an in-house MATLAB (Matlab 2014a, Mathworks, Natick, MA) script on an off-line workstation (Windows 7, Core i7-4930, 128GB RAM).

### Image evaluation

The reconstructed images were evaluated both quantitatively and qualitatively. For quantitative image evaluation, several masks were predefined by image processing of the SoS data and used.

#### Image masks

In order to limit the evaluation of an image to regions that are clinically relevant in TOF-MRA, several masks were defined and used for the quantitative analysis (Fig 2).

1. Brain mask: The brain mask was designed to overlie the brain so as to exclude the skull and extra-cranial regions. This was obtained by an automatic brain contour extraction tool (BET)[14].
2. Arterial mask: The arterial mask was designed to specifically overlie the cerebral arteries. The cerebral arteries were extracted by using the WEKA trainable segmentation tool available in the Fiji image processing package[15].
3. Brain parenchymal mask: The brain parenchymal mask was designed to overlie the brain parenchyma, which corresponds to the background of the arteries. It was created by subtracting the arterial mask from the brain mask.

### Quantitative image evaluation

The quantitative metrics comprised three groups; (1) simple image similarity metrics, namely the NMSE and the PSNR, (2) similarity metrics that try to simulate human perception, namely the SSIM and the scale invariant feature transform (SIFT), and (3) the contrast-to-noise ratio (CNR) between the cerebral arteries and the brain parenchyma.

The NMSE / PSNR / SSIM and SIFT were calculated for the maximum intensity projection (MIP) of the reconstructed images in the craniocaudal direction and also for the MIP images after applying the brain mask. The CNR was calculated by applying the arterial mask and the parenchymal mask.

### NMSE / PSNR / SSIM

NMSE is a simple image metric that is defined as an average of the squared intensity differences of two images, normalized by the reference image[16]. PSNR is the ratio between the maximum possible value (power) of an image and the power of distorting noise that affects the quality of its representation[16]. In this study, they are calculated as follows:

$$MSE(x,y)=\frac{1}{nm}\sum_{i=0}^{m-1}\sum_{j=0}^{n-1}{\left[x(i,j)-y(i,j)\right]}^{2}$$

$$NMSE(x,y)=\frac{MSE(x,y)}{MSE(x,0)}$$

$$PSNR(x,y)=10\;{\text{log}}_{10}\frac{{MAX}^{2}}{MSE(x,y)}$$

where *x* is the gold standard image, *y* is the reconstructed image and MAX is the maximum possible pixel value of the images. Both *x* and *y* are craniocaudalMIP images with a resolution of *m*×*n* pixels.

The SSIM index measure works by measuring the structural similarity that compares local patterns of pixel intensities that have been normalized for luminance and contrast[17]. It is based on the idea that the human visual system is good at extracting information based on structure. Local SSIM values for a window size of 8×8 pixels are calculated for every pixel of the image with the following formula:

$$SSIM(x,y)=\frac{\left(2\mu_{x}\mu_{y}+c_{1})(2\sigma_{xy}+c_{2}\right)}{\left(\mu_{x}^{2}+\mu_{y}^{2}+c_{1})(\mu_{x}^{2}+\mu_{y}^{2}+c_{2}\right)}$$

where *x* and *y* are the local windows for the reference image and reconstructed image respectively, *μ*_*x*_ and *μ*_*y*_ are the averages of *x* and *y* respectively, *σ*_*x*_ and *σ*_*y*_ are the variances of *x* and *y* respectively, and *σ*_*xy*_ is the covariance of *x* and *y*. c_1_ and c_2_ are parameters that stabilize the division with small denominator, determined arbitrarily. The SSIM index is the mean of all local SSIM values calculated by the formula above.

### CNR of artery-to-parenchyma

The CNR of artery-to-parenchyma was defined as the ratio of the signal difference between the artery and the parenchyma against the signal fluctuation of the background structure, which in our study is estimated by the standard deviation (SD) of the brain parenchyma, with the following formula:

$$\text{CNR}=({\text{S}}_{\text{artery}}-{\text{S}}_{\text{parenchyma}})/{\text{SD}}_{\text{parenchyma}}$$

S_artery_ and S_parenchyma_ are the mean signal intensities of the cerebral arteries and parenchyma of the reconstructed image respectively, and SD_parenchyma_ is the estimate of noise. However, instead of using the SD of the reconstructed image, the SD of the subtracted image, i.e. the subtraction image of the reconstructed image from the reference standard image, was used. This gives a better estimate of the noise generated in the CS reconstruction process. S_artery_, S_parenchyma_ and SD_parenchyma_ were calculated by applying the arterial mask and parenchymal mask (Fig 2).

#### SIFT

SIFT is a scale and rotation invariant feature descriptor that is highly effective in detection of local similarity between images[18]. In SIFT, two images are first detected for multiple key locations in scale space by looking for locations that are maxima or minima of a difference-of-Gaussian function. For each key location a local feature vector called the descriptor is calculated, which is invariant of rotation and scale and is minimally affected by noise and distortion. The descriptors of the two images are matched by an efficient nearest-neighbor search algorithm to find local similarities.

In our study, a list of key locations and descriptors were first calculated for the reference standard MIP image after applying the brain mask. The descriptors of the reconstructed MIP image with the brain mask were then calculated for the same key locations as the reference image, thereby ensuring that the corresponding areas were compared. Finally the mean of Euclidian distances of these pairs of descriptors was calculated as the indicator of similarity; i.e. the smaller value the more similar. The vlfeat open source library was used for this calculation[19]. All parameters were set to the default values.

**Fig 3:**
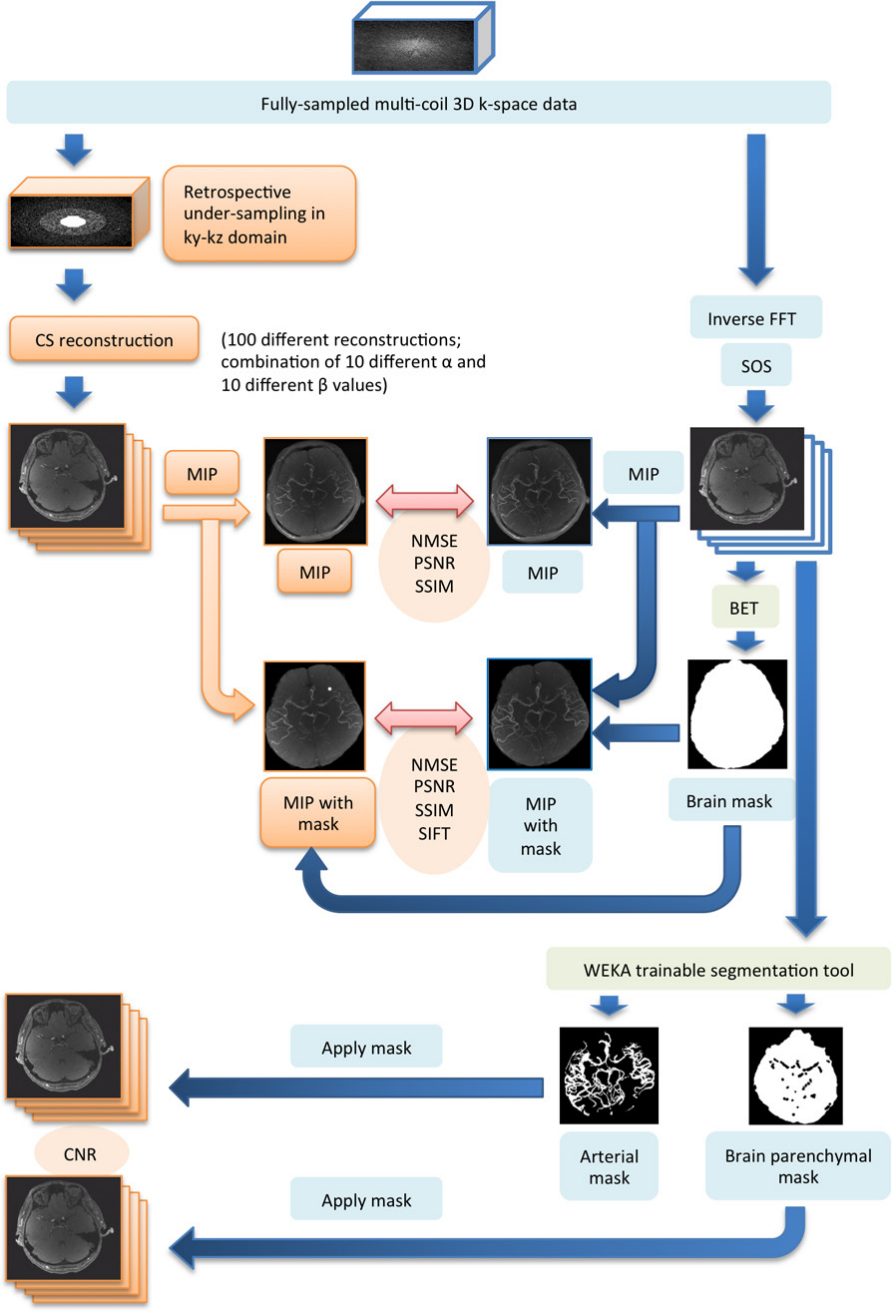
Flowchart of the overall image processing and calculation workflow for all image quality metrics.

Fig 3 is a flowchart of the image processing and calculation workflow for each image quality metric described above.

### Qualitative image evaluation

Visual evaluation was performed by two board-certified radiologists with clinical experience of 8 and 14 years, respectively. The reconstructed MIP images were rated on a scale from 1 to 5 (1; poor, 2; better but clinically unacceptable, 3; clinically acceptable, 4; clinically acceptable and good, 5; excellent) by focusing on the visibility of relatively small vessels. A score was determined for each image by consensus of the two radiologists. The best image was also chosen under the same criterion.

## Results

The image reconstruction with FCSA took approximately 8 minutes on average for 15 iterations. The SIFT algorithm detected 121 key locations from the reference standard MIP image.

### Visual evaluation

**Fig 4:**
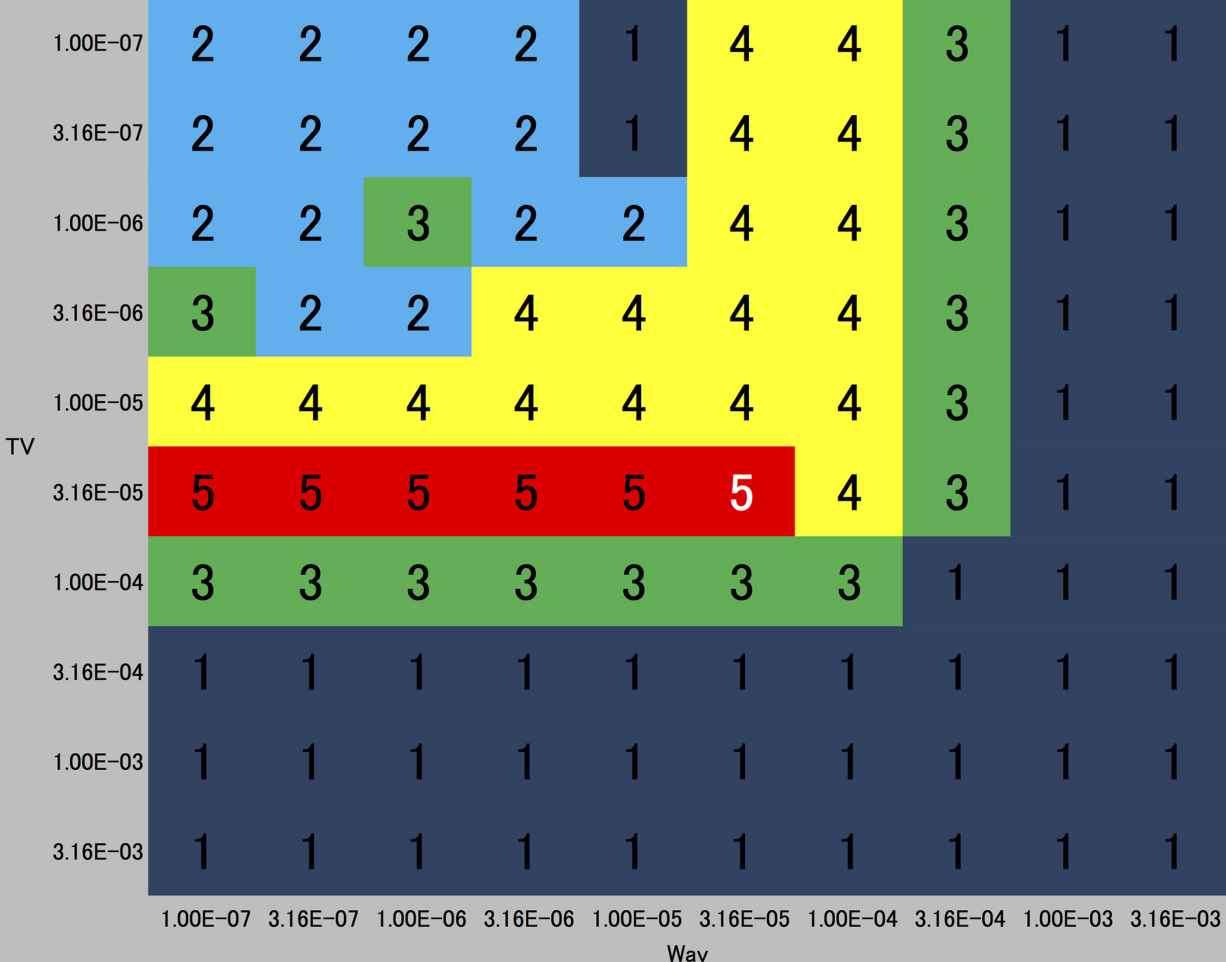
Result of the visual evaluation by consensus of two radiologists. The axes are wavelet coefficient (α) and total variation (TV) coefficient (β). The score (1 to 5) is denoted on each square. The highest ratings concentrate at β = 3.2 × 10^−5^ and the parameter pair that reconstructed the best image (α = 3.2 × 10^−5^, β = 3.2 × 10^−5^) is denoted as a number in white.

**Fig 5:**
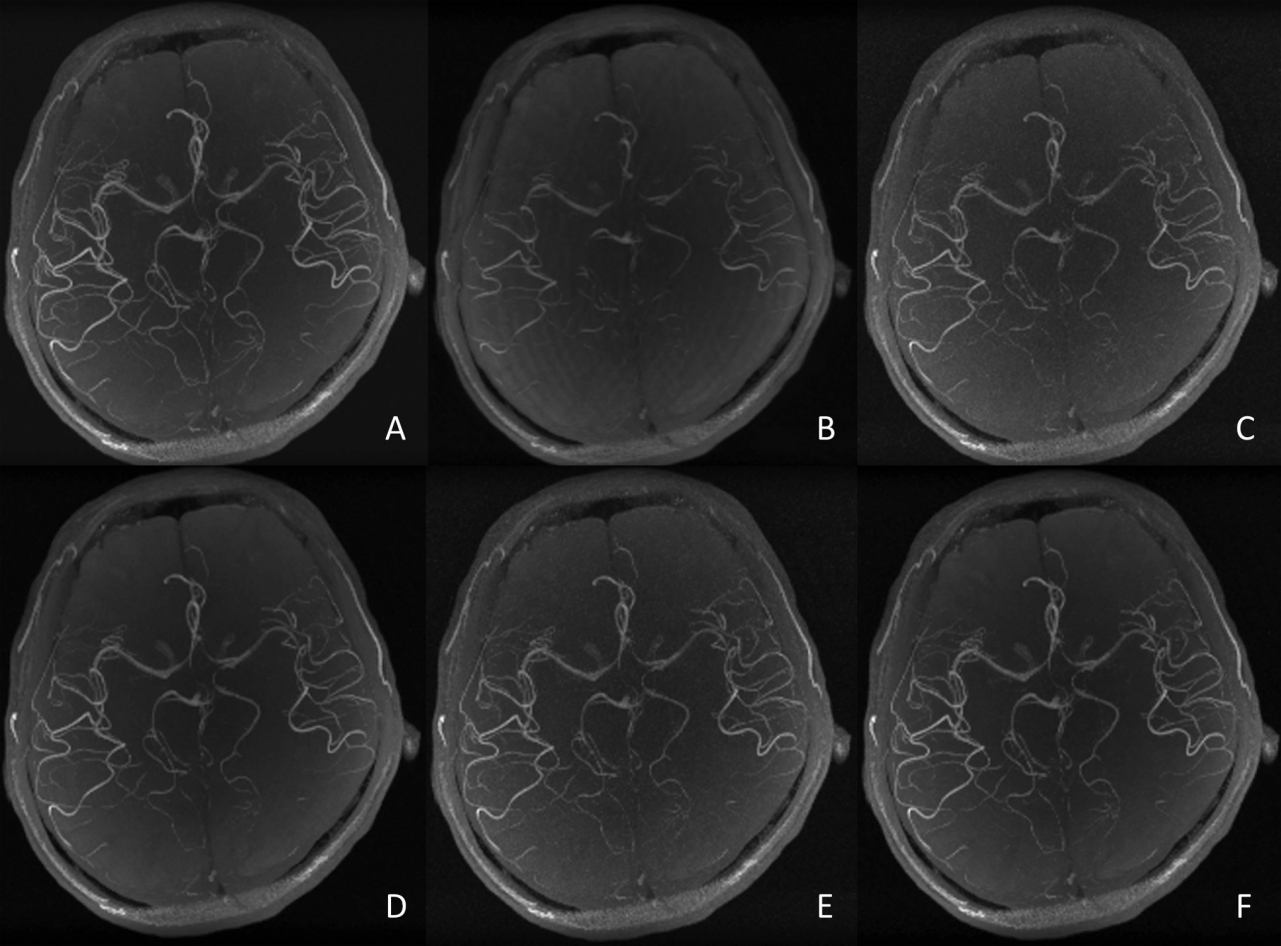
Craniocaudal maximum intensity projections of reconstructed magnetic resonance angiography. (A) SoS image and sample images from a visual rating of (B) 1 (α = 1.0 × 10^−4^, β = 3.2 × 10^−3^), (C) 2 (α = 1.0 × 10^−7^, β = 1.0 × 10^−6^), (D) 3 (α = 3.2 × 10^−5^, β = 1.0 × 10^−4^), (E) 4 (α = 1.0 × 10^−6^, β = 1.0 × 10^−5^) and (F) 5 (α = 3.2 × 10^−5^, β = 3.2 × 10^−5^).

The visual ratings of the MIP images are shown in Fig 4. A sample image of each rating is shown in Fig 5. As can be seen, the small arteries in the temporal (M2, insular segment of middle cerebral artery (MCA)) and posterior lobes (P2, ambient segment, P3, quadrigeminal segment of posterior cerebral artery) are more discernible in the higher rated images, whereas lower signal intensity of the small arteries (M1, sphenoidal segment of MCA, M2, P2, P3) and more noise in the background are evident in the lower rated images. The regularization parameter values of the CS reconstruction with the highest visual score (i.e., 5 out of 5) are denoted in red, which are *α* = 1.0 × 10^−7^ to 3.2 × 10^−5^ and *β* = 3.2 × 10^−5^. Among these parameter values, as the value of *α* decreases, the signal intensities of the small arteries (M2, P2, P3) slightly increase but more noise is seen in the background. This is a trade-off in terms of visibility of the arteries. The best image chosen in the visual evaluation was that reconstructed by a parameter pair of (*α*,*β*) = (3.2 × 10^−5^, 3.2 × 10^−5^).

### Statistical evaluation

**Fig 6:**
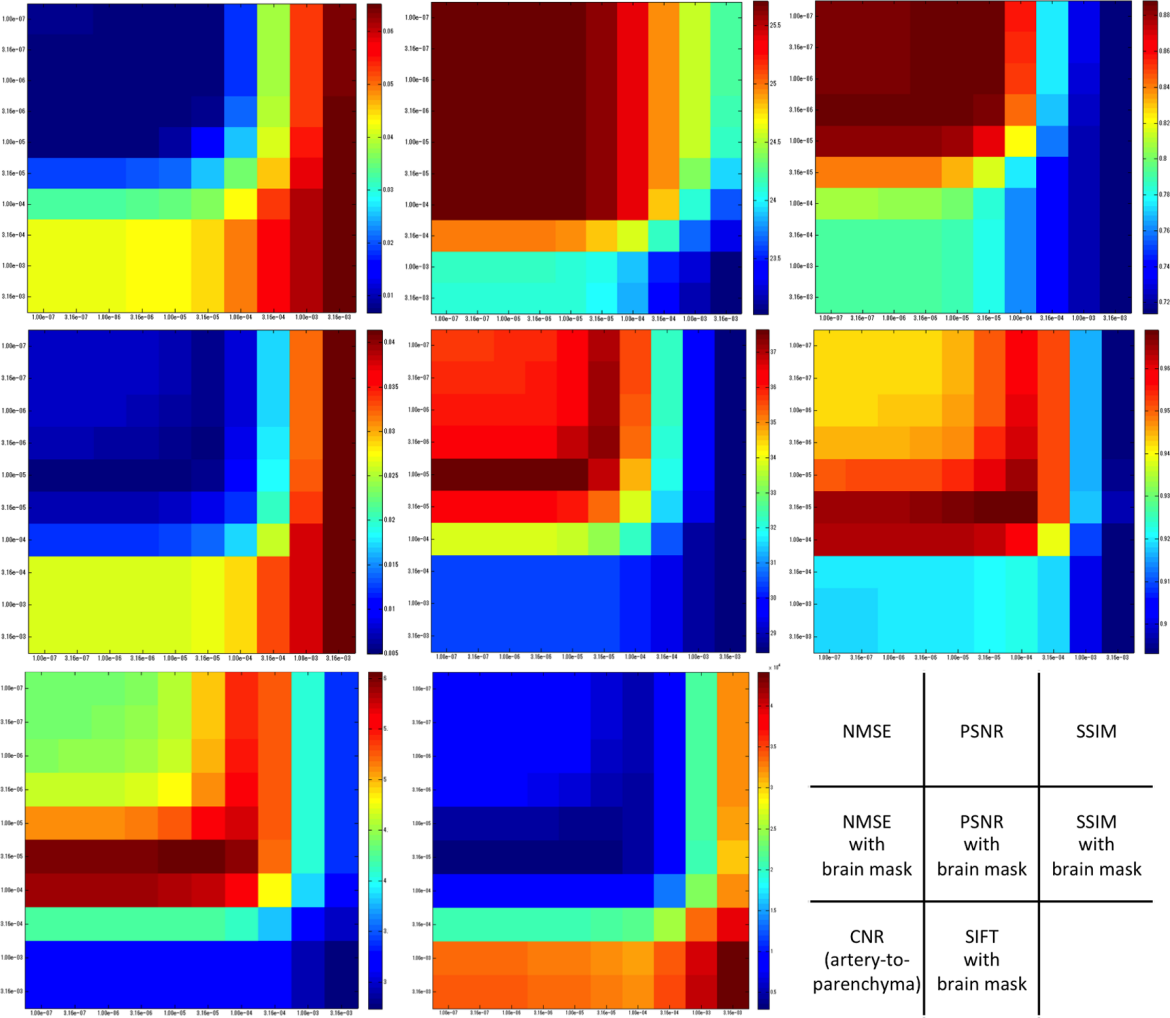
Color plots of all metrics against the regularization parameter pairs of total variation (α) and wavelet coefficients (β). The vertical axis of each plot is α (1.0 × 10^−7^ to 3.2 × 10^−3^ from top to bottom) and the horizontal axis is β (1.0 × 10^−7^ to 3.2 × 10^−3^ from left to right). For NMSE and SIFT, lower values reflect better results (i.e. in blue), and vice versa for PSNR, SSIM and CNR (i.e. good results in red). Note the similarity and dissimilarity with visual evaluation (Fig 4).

**Fig 7:**
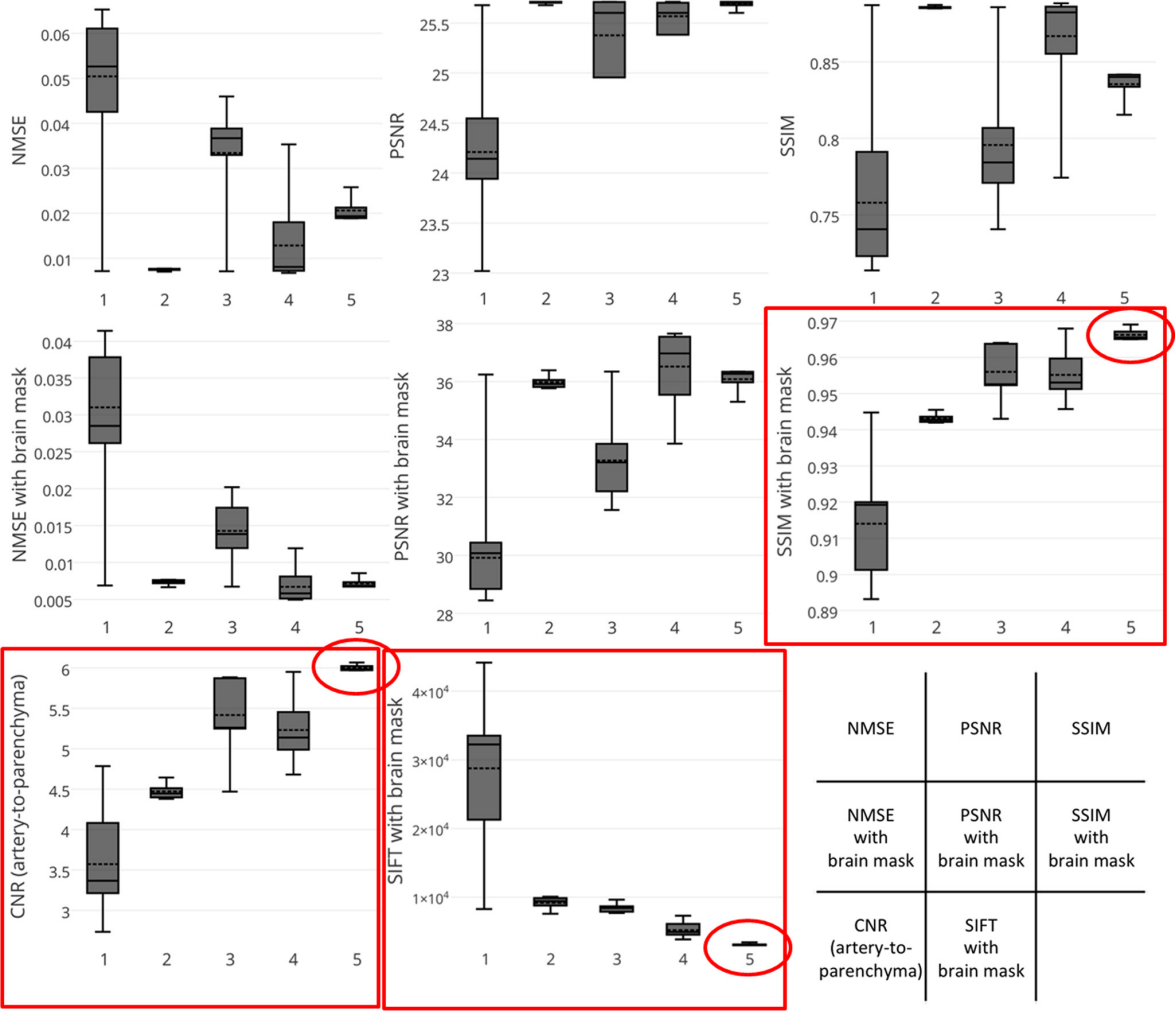
Box-whisker plots of all metrics (vertical axis) against the visual score (horizontal axis). The red boxes and circles indicate how SSIM and SIFT with brain mask and CNR of artery-to-parenchyma show good correlation with the visual rating, especially with the highest rated images (i.e. score of 5) which are most relevant.

**Fig 8:**
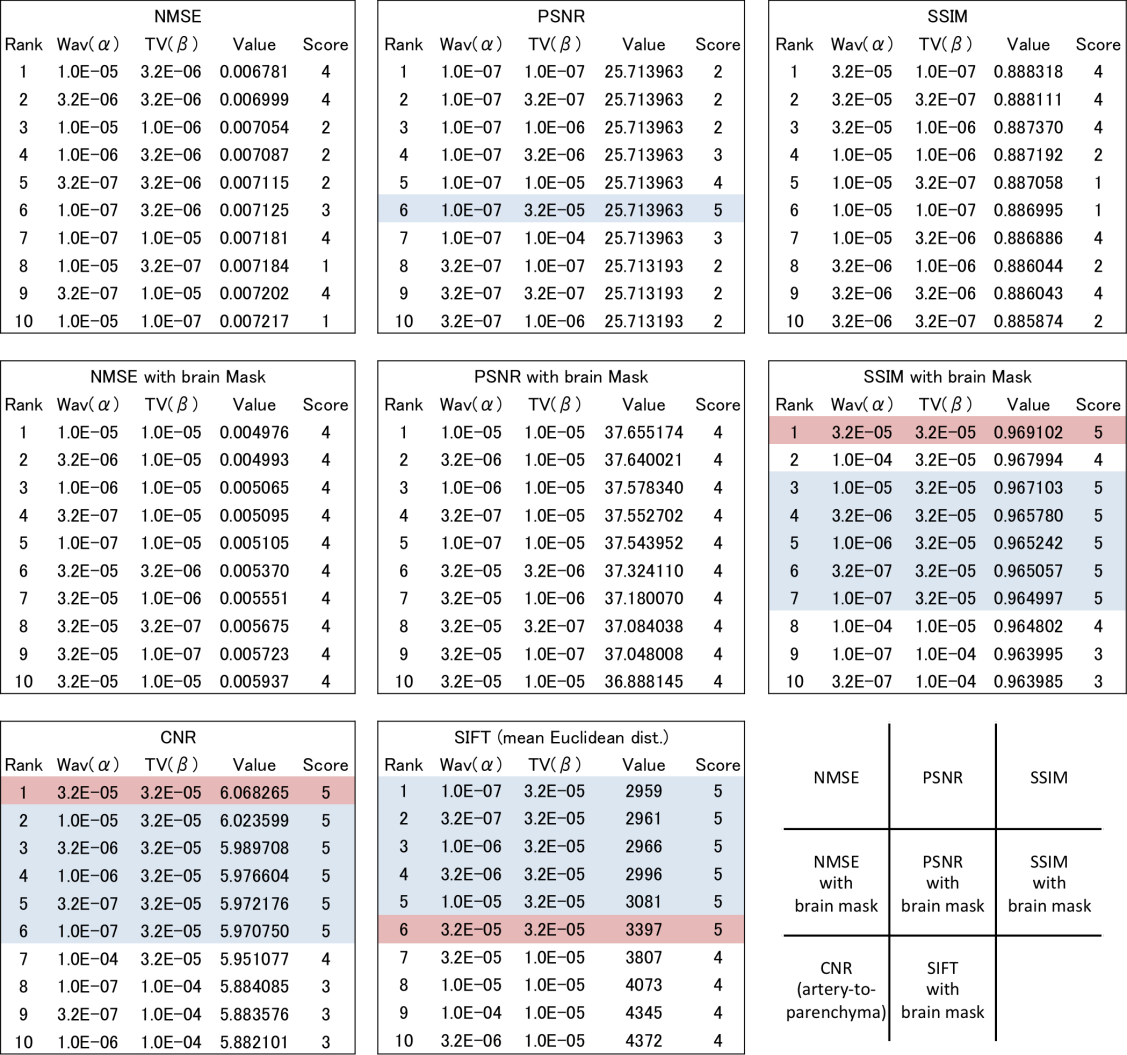
The top 10 values for each metric. The parameters that generated the visually best image are noted in red and the highest score in blue. The SSIM with brain mask and CNR have ranked the visually best image (reconstructed with α = 3.2 × 10^−5^ and β = 3.2 × 10^−5^) on the top. CNR and SIFT have ranked the six highest rated images in the top six and SSIM with brain mask ranked the six highest rated images in the top seven.

The results of the quantitative measures are plotted as color maps against the values of *α* and *β* (Fig 6). Box-whisker plots for the statistical measures considered against the visual ratings are shown (Fig 7). The parameter pairs (*α*,*β*) which yielded the top-10 values of each statistical measure are summarized in Fig 8. For reference, a color plot of the average slice-by-slice NMSE values are included as supporting information (S1 Fig).

## Discussion

Generally, optimization of regularization parameters in CS cannot be automated because it requires knowledge about the optimal results. The optimal parameter highly depends on the observation model, as well as the object of interest itself. Therefore, there is no definitive approach for optimization of regularization parameters. In the case of CS in TOF-MRA, quantification of similarity with statistical metrics such as NMSE and PSNR between the reconstructed image and the reference standard image have been tried[20,21].

In our study, conventional distance metrics such as NMSE and PSNR between the reconstructed image and the reference standard image did not reflect visual scores (Fig 4 and Fig 6). These simple error measures may not be appropriate because they do not necessarily correlate with visualization of peripheral arteries, and moreover the irrelevant structures such as brain parenchyma and skull skew the optimization process.

SSIM accounts for spatial correlation of coefficients at each voxel and can simulate human perception in structure. Nevertheless, this method may not be sufficient for evaluation of fine structures such as peripheral arteries. Another approach is the L-surface method which tries to balance data consistency and L1 penalty, but the determination of the balanced point is arbitrary and remains to be inconsistent.

NMSE, PSNR and SSIM are image quality metrics that are widely used to evaluate the performance of image reconstruction techniques. These metrics are useful for evaluation of phantom data, but cannot be easily applied to real data with abundant noise. Nevertheless, many of the previous studies assumed that these metrics correlate well with human perception[9,21,22].

In our study, these conventional statistical metrics alone demonstrated a tendency to choose smaller values for both *α* and *β* as superior images, which is depicted as a general color gradient from left-to-right and top-to-bottom (Fig 6). These metrics hardly demonstrated a correlation between visual and statistical evaluation. The reason for this could probably be attributable to the irrelevant extra-cranial structures such as skull, paranasal sinuses and air around the head.

Our results show that the metric that demonstrated the best performance was not NMSE, PSNR or SSIM alone but instead SSIM, SIFT and CNR with the application of adequate masks. As can be seen from Fig 8, SSIM and SIFT with brain mask, CNR with arterial and parenchymal masks all highly rated the images reconstructed with *β* = 3.2 × 10^−5^ and a relatively small value of *α*, ranging from 1.0 × 10^−7^ to 1.0 × 10^−4^. The best image in the visual evaluation, which was reconstructed by a parameter pair of (*α*,*β*) = (3.2 × 10^−5^, 3.2 × 10^−5^), was included in the top six evaluated by any of these three metrics. CNR of artery-to-parenchyma and SSIM with brain mask have ranked the visually best image on the top. SSIM and SIFT with brain mask have ranked the six visually highest rated images in the top six. SSIM with brain mask ranked the six images in the top seven. In short, the results of these metrics showed good concordance with qualitative evaluation by radiologists.

To our surprise, not only were SSIM with brain mask and CNR of artery-to-parenchyma able to select the visually highest scored images correctly, but they were also able to rank the best image (*α* = 3.2 × 10^−5^, *β* = 3.2 × 10^−5^) at the top. This is astonishing considering the subtle differences among the highest scored images. The apparent difference between these images is the slightly smooth noise in the background and thus slightly superior visibility of the fine arteries in the best image, while the other images (*α* = 1.0 × 10^−5^ to 1.0 × 10^−7^, *β* = 3.2 × 10^−5^) are almost indistinguishable. We believe it demonstrates the precision and similarity of these statistical metrics with human visual perception[23].

The CNR takes both contrast and noise into consideration and has been shown to accurately reflect tissue differentiation in MRI[24]. Of note is that the CNR is the only statistical measure in which all slices of the TOF-MRA image set were used in the calculation and also which does not require a reference standard; all other measures were comparisons of the reconstructed and reference standard MIP images. This may be one of the reasons why the CNR was able to select the best visual image despite the subtle differences in the visibility of the fine arteries.

Optimal regulation parameters may depend on MRI acquisition parameters. However, it is difficult to subjectively determine optimal regularization parameters for all possible MRI acquisition parameters. Our results indicate that these metrics have a potential to enable automated optimization of regularization parameters. There still needs further investigation into why the three different metrics (SIFT, SSIM and CNR) showed a similar behavior.

There have been some studies that incorporated region-of-interests (ROI) for improvement of image reconstruction in CS[20,25]. Since a mask is a type of ROI, these studies are strongly correlated with our study. By defining a structure of interest by a ROI, they have shown that the result of CS reconstruction can be improved, whereas we have shown that ROI can be effectively used for parameter optimization. We believe that the significance of our study lies in that the radiologists’ perception was directly correlated with objective measures. Our results demonstrate that statistical image metrics that are defined mathematically could serve as a practical indicator of radiologists’ perception, which to our knowledge has never been reported previously.

There were several limitations in our study. First, we have performed this study on only one patient’s data. In principle, the parameters determined in our study can be applied when scanning other patients as long as all other parameters and conditions are identical. Further studies with other patients need to be conducted in order to validate the usefulness of our result. Second, the visual evaluation and the computation of most of the statistical metrics were performed on the craniocaudal MIP images. In clinical practice, MIP images are usually created in a number of directions, e.g. a total of 12 images, each incremented by a rotation of 15 degrees. Therefore there may be a discrepancy between our results and clinical setting, although we believe this to be trivial since the effects of noise and image distortion by CS affects the entire scanned volume more or less uniformly. Third, several parameters in the computation of the metrics have been determined arbitrarily which attenuates the robustness and consistency of the study. Of note, there is some inconsistency in creating brain and arterial masks because a number of parameters need to be arbitrarily determined for BET and the WEKA trainable segmentation tool. In case the generated mask is suboptimal, the results can be quite unreliable, but it is very difficult to evaluate whether the generated arterial mask is optimal or not.

In conclusion, we have shown that certain image metrics, namely SSIM and SIFT in conjunction with a mask that overlies the brain, and CNR of artery-to-parenchyma, showed a good concordance with radiologists’ qualitative evaluation. By carefully selecting a region to measure, statistical image metrics can reflect radiologists’ visual evaluation, thus enabling an appropriate optimization of regularization parameters for CS.

## Supporting Information

S1 Fig A color plot and top 10 list of the average slice-by-slice NMSE values between the SoS and reconstructed images. The image to the left is a color plot of the average slice-by-slice NMSE values between the SoS and reconstructed images. The axes are the same as in Fig 6; the vertical axis is α (1.0 × 10^−7^ to 3.2 × 10^−3^ from top to bottom) and the horizontal axis is β (1.0 × 10^−7^ to 3.2 × 10^−3^ from left to right). Notice the dissimilarity with the result of visual evaluation (Fig 4).
(TIF)
