# Correction: Optimization of Regularization Parameters in Compressed Sensing of Magnetic Resonance Angiography: Can Statistical Image Metrics Mimic Radiologists' Perception?

**DOI:** 10.1371/journal.pone.0197140

**Published:** 2018-05-07

**Authors:** Thai Akasaka, Koji Fujimoto, Takayuki Yamamoto, Tomohisa Okada, Yasutaka Fushimi, Akira Yamamoto, Toshiyuki Tanaka, Kaori Togashi

The fifth author’s name is spelled incorrectly. The correct name is: Yasutaka Fushimi. The correct citation is: Akasaka T, Fujimoto K, Yamamoto T, Okada T, Fushimi Y, Yamamoto A, et al. (2016) Optimization of Regularization Parameters in Compressed Sensing of Magnetic Resonance Angiography: Can Statistical Image Metrics Mimic Radiologists' Perception? PLoS ONE 11(1): e0146548. https://doi.org/10.1371/journal.pone.0146548
