# Supplementary figures and images for: Optimization of Regularization Parameters in Compressed Sensing of Magnetic Resonance Angiography: Can Statistical Image Metrics Mimic Radiologists' Perception?
Source: PLoS One. 2016 Jan 8;11(1):e0146548. doi: 10.1371/journal.pone.0146548 (PMC4706324; doi:10.1371/journal.pone.0146548)

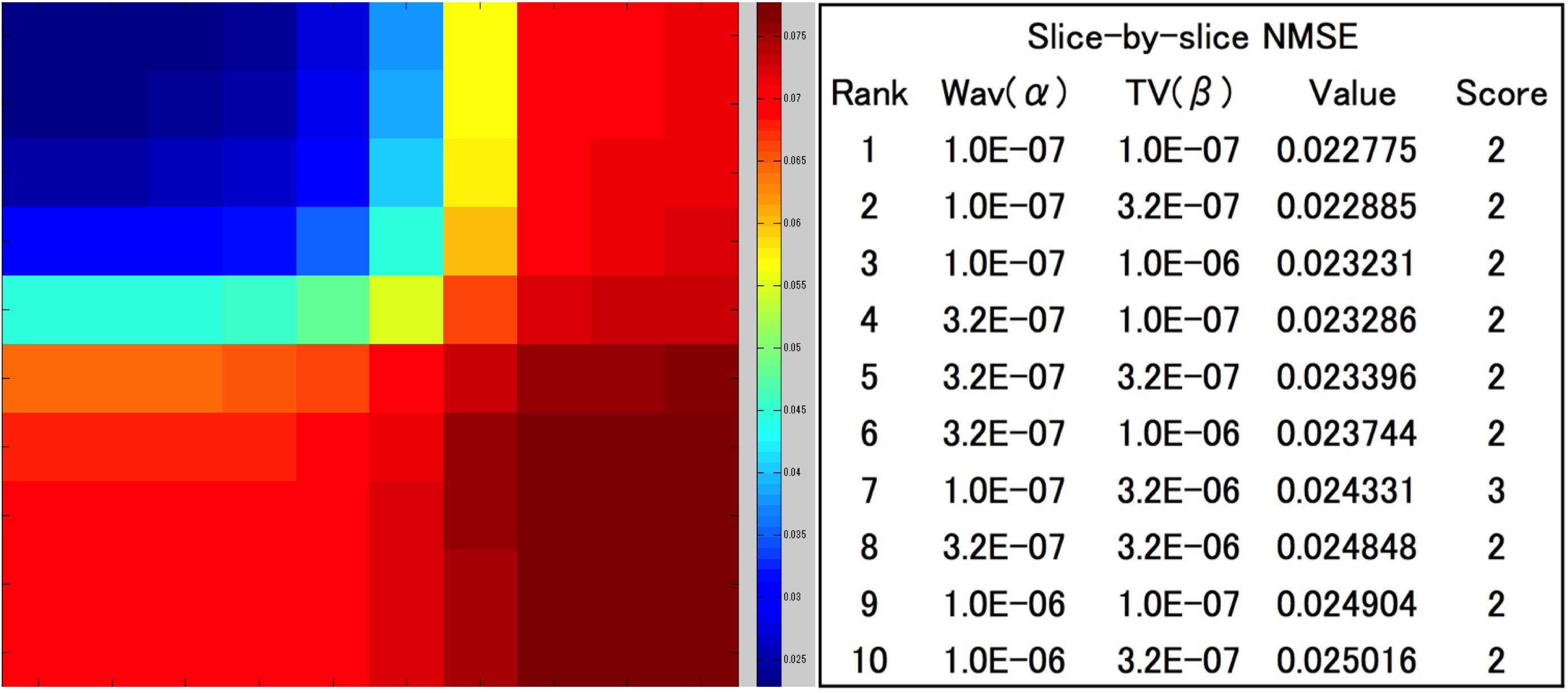

Supplement: S1 Fig — The image to the left is a color plot of the average slice-by-slice NMSE values between the SoS and reconstructed images. The axes are the same as in Fig 6; the vertical axis is α (1.0 × 10^−7^ to 3.2 × 10^−3^ from top to bottom) and the horizontal axis is β (1.0 × 10^−7^ to 3.2 × 10^−3^ from left to right). Notice the dissimilarity with the result of visual evaluation (Fig 4). (TIF) [file pone.0146548.s001.tif]
